# Anilinoquinazoline inhibitors of the RET kinase domain—Elaboration of the 7-position

**DOI:** 10.1016/j.bmcl.2016.03.100

**Published:** 2016-06-01

**Authors:** Allan M. Jordan, Habiba Begum, Emma Fairweather, Samantha Fritzl, Kristin Goldberg, Gemma V. Hopkins, Niall M. Hamilton, Amanda J. Lyons, H. Nikki March, Rebecca Newton, Helen F. Small, Swamy Vishwanath, Ian D. Waddell, Bohdan Waszkowycz, Amanda J. Watson, Donald J. Ogilvie

**Affiliations:** a Drug Discovery Unit, Cancer Research UK Manchester Institute, University of Manchester, Wilmslow Road, Manchester M20 4BX, UK; b Medicinial Chemistry, SAI Life Sciences, Ltd, Pune, India

**Keywords:** Kinase inhibitor, RET receptor, Hepatocyte stability, Targeted therapy, Lung cancer

## Abstract

We have previously reported a series of anilinoquinazoline derivatives as potent and selective biochemical inhibitors of the RET kinase domain. However, these derivatives displayed diminished cellular potency. Herein we describe further optimisation of the series through modification of their physicochemical properties, delivering improvements in cell potency. However, whilst cellular selectivity against key targets could be maintained, combining cell potency and acceptable pharmacokinetics proved challenging.

## Graphical abstract

**Figure:**
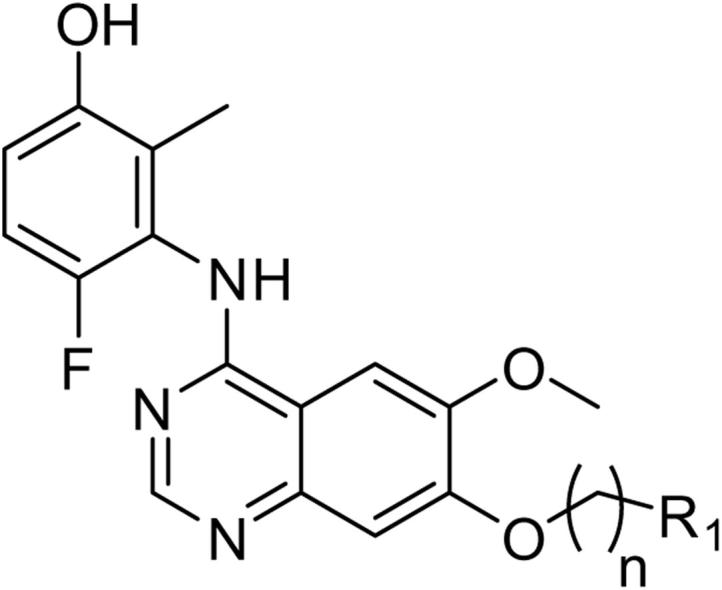

Recent advances in genomic sequencing technology have facilitated unprecedented analysis of tumour DNA, allowing patient stratification based not on histopathology, but on the key driver mutations found within the tumour itself. This approach has led to dramatic responses in disease settings where standard therapies have proved ineffective. Examples of success in this area include malignant melanoma, where the V600E mutant form of B-Raf can be inhibited with vemurafenib (Zelboraf™) **1**,^1^ and mtEGFR, which can be inhibited with gefitinib (Iressa™)^2^ **2** (Fig. 1).

However, activating mutations form only a subset of driver oncogenes and there is increasing recognition that certain cancer subtypes are driven by gene fusion products where constitutive activation of specific kinase domains results in over-active downstream signalling.^3^ For example, the EML4-ALK fusion present in 1–2% of lung cancers leads to uncontrolled proliferation through activation of the MAPK, PI3K/AKT and JAK3/STAT3 pathways.^4^ Blockade of this signalling with crizotinib (Xalkori™) **3**,^5^ a re-purposed inhibitor originally designed as a Met kinase inhibitor, has resulted in dramatic clinical responses^6^ and estimated net sales of >$280 M in 2013.^7^

More recently, evidence has emerged that gene fusion products incorporating the tyrosine kinase domain of the RET receptor can also provide the oncogenic drive required to initiate and sustain tumour growth.^8^ Initially identified in around 1–2% of non-small cell lung cancer cases, this fusion is now reported to occur in a diverse range of tumour types, including pancreatic, colon and breast cancers.^9^, ^10^

The currently available non-selective inhibitors of the RET kinase domain, such as cabozantinib (Cometriq™) **4**,^11^ ponatinib (Iclusig™) **5**,^12^ and vandetanib (Caprelsa™) **6**^13^ are far from ideal.^14^, ^15^ Serious adverse side effects limit their utility in the chronic setting and, in some cases, have led to treatment-related patient deaths. Several of these effects appear to stem from the potent inhibition of the VEGFR-2 kinase, also known as KDR. Given this, we believe that there is a clear clinical need for potent, drug-like and selective RET inhibitors which do not display off-target pharmacological inactivation of KDR.

In an effort to address this need, we recently reported a series of anilinoquinazolines which featured derivatives of a 3-hydroxyanilino head group.^16^, ^17^ Derivatives such as compound **7**, bearing an *ortho*-methyl substituent, delivered strong enzymatic potency and surprising selectivity against KDR but were limited by a considerable reduction in cellular activity against the RET kinase domain and, as a consequence, compressed selectivity against KDR in the cellular context.

**Figure:**
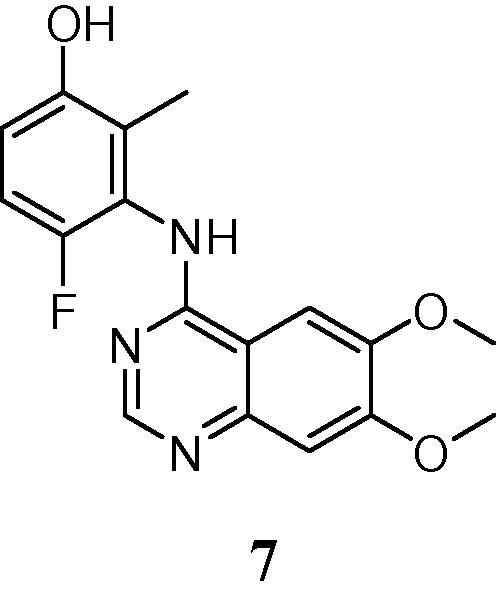

Moreover, these derivatives were limited by metabolic instability, likely due to the phenol moiety, which precluded their further exploration. Our previous studies in this area had indicated derivatives of this type to be comparatively stable in microsomal metabolism assays but considerably less so in the presence of hepatocytes,^16^ indicating that the compounds were susceptible to Phase II metabolic processing such as glucuronidation or sulfation, which is not uncommon or phenol moieties.^18^ Herein, we describe our further efforts in this area to improve both cellular potency and metabolic stability.

Our initial efforts focused upon attempts to find a bioisosteric replacement for the phenol that would maintain the key hydrogen bonding interaction observed in our original derivatives. (Fig.2a). In common with related kinase inhibitors, the quinazoline ring system binds to the hinge region of the ATP-binding site, with the anilino group buried in the so-called “selectivity” pocket above the gatekeeper residue Val-804. The crystal structure of the simple phenol derivative reveals a pair of hydrogen bonds from the phenol to Asp-892 (of the DFG loop) and Glu-775 (the conserved C helix glutamate).^16^ In this binding mode, the close contact of the 2-methyl against Ser-891 may be responsible for the enhanced biochemical selectivity over KDR, which has a larger cysteine residue in this position. As such, the targeted bioisosteres were selected in order to maintain this steric constraint, alongside maintenance of the desired hydrogen bonding network.

During this investigation, more than 30 examples of classical and non-classical bioisosteric replacements were prepared but these extensive studies revealed the SAR in this area to be extremely tight. Selected examples from this work are shown in Table 1 (Compounds **9**–**18**). Of the derivatives prepared, only the indazoles **16** and **18** retained sub-micromolar enzymatic activity against RET and of these two derivatives, only **18** demonstrated acceptable selectivity against KDR at the biochemical level. The observed SAR is in agreement with the proposed binding mode of these bicyclic systems, where only indazole **18** is capable of forming both of the hydrogen bonds observed crystallographically for the phenol (Fig.2b). The use of indazole as a phenol mimic in a similar binding context has been reported previously, e.g., for inhibitors of EphB4 tyrosine kinase.^19^ However, the fall in biochemical potency between **7** and **18** in this instance is presumably due to the larger bulk of **18** being disfavoured in the restricted binding pocket.

Disappointingly, biochemical potency and selectivity did not translate into the cellular setting, most likely due to modest permeability and high efflux (3.7 × 10^-6^ cm s^-1^, efflux ratio 17) which precluded **18** from further investigation. Moreover, this derivative demonstrated a somewhat shorter half-life in human liver microsomes compared to the parent compound **7** (124 min vs 223 min).

Since bioisosteric replacement of the phenol did not did not deliver the required attributes to allow an in vivo evaluation of pharmacokinetic parameters, our attention turned to the optimisation of the pendant functionality borne by the anilinoquinazoline scaffold itself.

Our inspiration in this area was partly drawn from a recently reported series of inhibitors of KDR, where a similar substantial increase in enzyme inhibitory potency was observed from the introduction of a meta-anilinophenol warhead to give lead compound **19**.^20^ However, in common with our own chemotype this derivative also suffered from extensive hepatic glucuronidation and resultant poor pharmacokinetics. Further optimisation revealed that the addition of a pendant moiety bearing a basic centre (as exemplified by **20**) not only improved overall pharmacokinetics, but also delivered a dramatic reduction in the extent of Phase I and Phase II metabolism, simultaneously increasing both stability in liver microsomes and resistance to glucuronidation.^21^ We hypothesized that a similar approach may offer comparable benefits in our lead series.

**Figure:**
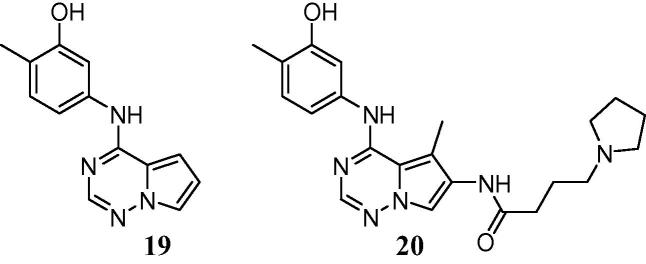

Our previous work on this template had demonstrated that substitution at the 7-position was more readily tolerated than at the 6-position of the quinazoline core, which tended to result in diminished potency and selectivity versus KDR at the biochemical and cellular levels (data not shown). Our investigations therefore focused upon this more favourable substituent pattern. Data representative of the >100 derivatives synthesized and tested are given in Table 2.

From a diverse series of substituents exploring the incorporation of a pendant basic centre, some early trends became evident. For basic substituents, a three carbon linker chain appeared to be generally preferable for both potency and, to an extent, selectivity (e.g., **26** vs **25**, **30** vs **29**). However, this trend was not universal and was dependent upon the specific nature of the tail group. Modelling of these derivatives suggests that the basic tails should extend towards the mouth of the ATP-binding site and into solvent, and, as indicated by molecular dynamics simulations, are more likely to form water-mediated interactions with the protein rather than direct hydrogen-bonding contacts (Fig. 3). This is in general agreement with the data in Table 2, where at best only modest gains in activity are observed for the basic groups compared with **7** or the extended ethers **49** and **50**.

The observed preference for longer carbon chains may be a consequence of an entropic gain due to greater conformational flexibility, with the bulky terminal moiety positioned further from the protein and more readily solvated. It is also noted that the amines in Table 2 represent a wide range of basicities, a feature which is also dependent on the carbon chain length (i.e., the predicted p*K*_a_ is higher for amines separated from the oxygen linker by three carbon atoms rather than two). Given that RET and KDR both contain several ionisable amino acid side-chains around the mouth of the pocket, long range electrostatic interactions may have some impact on potency and selectivity even in cases where no formal hydrogen-bonding contacts are formed.

Whilst we were pleased to find that many derivatives displayed potencies and selectivity comparable to, or better than, the parent derivative **7**, it was disappointing that this activity tended not to translate to the cellular environment, with many compounds failing to display potency in this more relevant context. Given the biochemical assay was conducted at an ATP concentration close to *K*_m_ for the RET kinase domain, we had anticipated a much closer correlation between the enzyme and cellular assays and we therefore attributed this disconnect to be due to limited permeability of these compounds, or potential differences in the binding conformation of the kinase domain in the biochemical and cellular assay systems.^16^

Indeed, most of the basic derivatives were found to be inactive (>10 μM) in our KDR cellular selectivity assay, though seven of the prepared derivatives displayed cell activity against RET comparable to the parent derivative. Amongst the latter compounds, only the trifluoromethylpiperazine derivative **43** displayed a significant improvement in activity and was the only compound found to display sub-micromolar RET activity in our cell-based systems.

To test the hypothesis that polar functionality, rather than a basic centre, may also improve metabolic stability whilst enhancing cellular permeability, a limited number of non-basic derivatives, exemplified by **48**–**50**, were also prepared. These derivatives tended to show similar potencies and selectivities to the basic derivatives when tested in our biochemical assay, but tended to maintain activity in the cellular systems, giving data comparable to **7**. This finding, along with the data for **43**, indicates that permeability and physicochemical parameters may be a limiting factor for these derivatives and that further modification of both the p*K*_a_ of the tailgroup and the overall Log *P* of the derivative may potentially allow further improvement in this area.

To investigate whether these present modifications had mitigated the metabolic deficiencies experienced with the phenol warhead, the seven most cell-active compounds were investigated in in vitro human metabolic stability assays (Table 3). Whilst **49** was exceptionally stable in a microsomal (Phase I) metabolic assay, most compounds failed to show an improvement relative to compound **7**. However, an interesting disconnect in stability was observed between microsomal stability and stability in hepatocytes, where both Phase I and II metabolism occurs. Whilst some compounds, such as **44**, behaved similarly to the parent compound **7** and showed diminished stability in hepatocytes compared to microsomes (as may be anticipated for a phenolic moiety), we were pleased to find that compounds **41**, **48** and **50** were observed to be relatively metabolically stable.

Incorporation of a pendant, basic tail group has previously been demonstrated to deliver an improvement in phenol metabolism. In this setting, this strategy does not appear to be universally beneficial to mitigate hepatic metabolism. Whilst derivatives such as compound **43** displayed improved cell potency compared to the parent derivative, concomitant improvements in metabolic stability were not generally observed. However, compounds such as **41**, **48** and **50** maintained cellular potency and selectivity whilst showing some improvement in the overall metabolic profile of these agents. These data suggest that further improvements to this series, through modification of physicochemical properties, may offer additional potential for improvements in both metabolic stability and cellular potency.

The described derivatives were synthesized according to the following schemes. As detailed in Scheme 1, the commercially available chloroquinazoline **51** was functionalized through an S_N_Ar displacement of the 4-chloro moiety with the required aromatic amine to yield the desired bioisosteres **8**–**18**.

However, the majority of the derivatives for this study were prepared as described in Scheme 2. The previously described imine adduct **52**^22^ was readily alkylated with either 1-chloro-2-bromoethane or 1-chloro-3-bromopropane to yield intermediates **53** and **54**. Cyclisation with the requisite anilinophenol^16^ yielded the advanced chloroalkyloxy anilinoquinazolines **55** and **56** in moderate yield. These alkyl halides could then be further elaborated without intermediate purification simply by S_N_2 alkylation with the desired amine, and the target compounds purified by preparative HPLC, to yield **21**–**44** and **50**.

Certain derivatives, particularly those with cyclic or acyclic ethers, were more conveniently synthesized using the method detailed in Scheme 3. The known mono-methylated chloroquinazoline **57**^23^, ^24^ was functionalized using Mitsonobu methodology to install the desired pendant functionality at the 7-position, followed by an S_N_Ar displacement of the 4-chloro moiety with the required aniline^16^ to yield **45**–**49**.

## Figures and Tables

**Figure 1:**
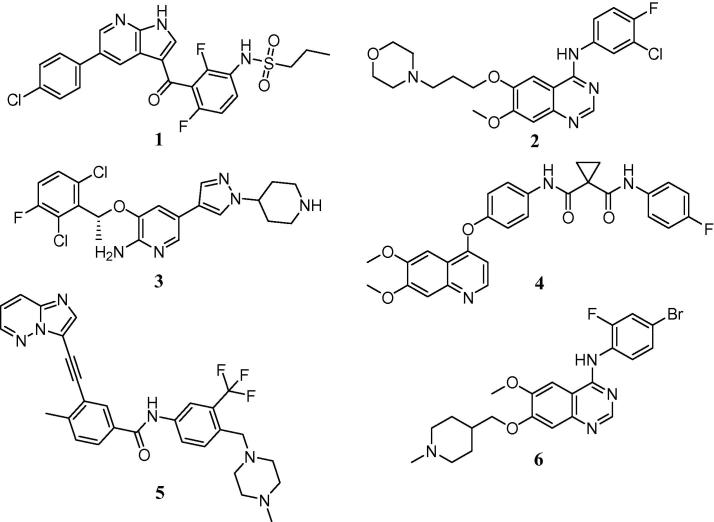
Exemplar targeted therapeutic agents.

**Figure 2:**
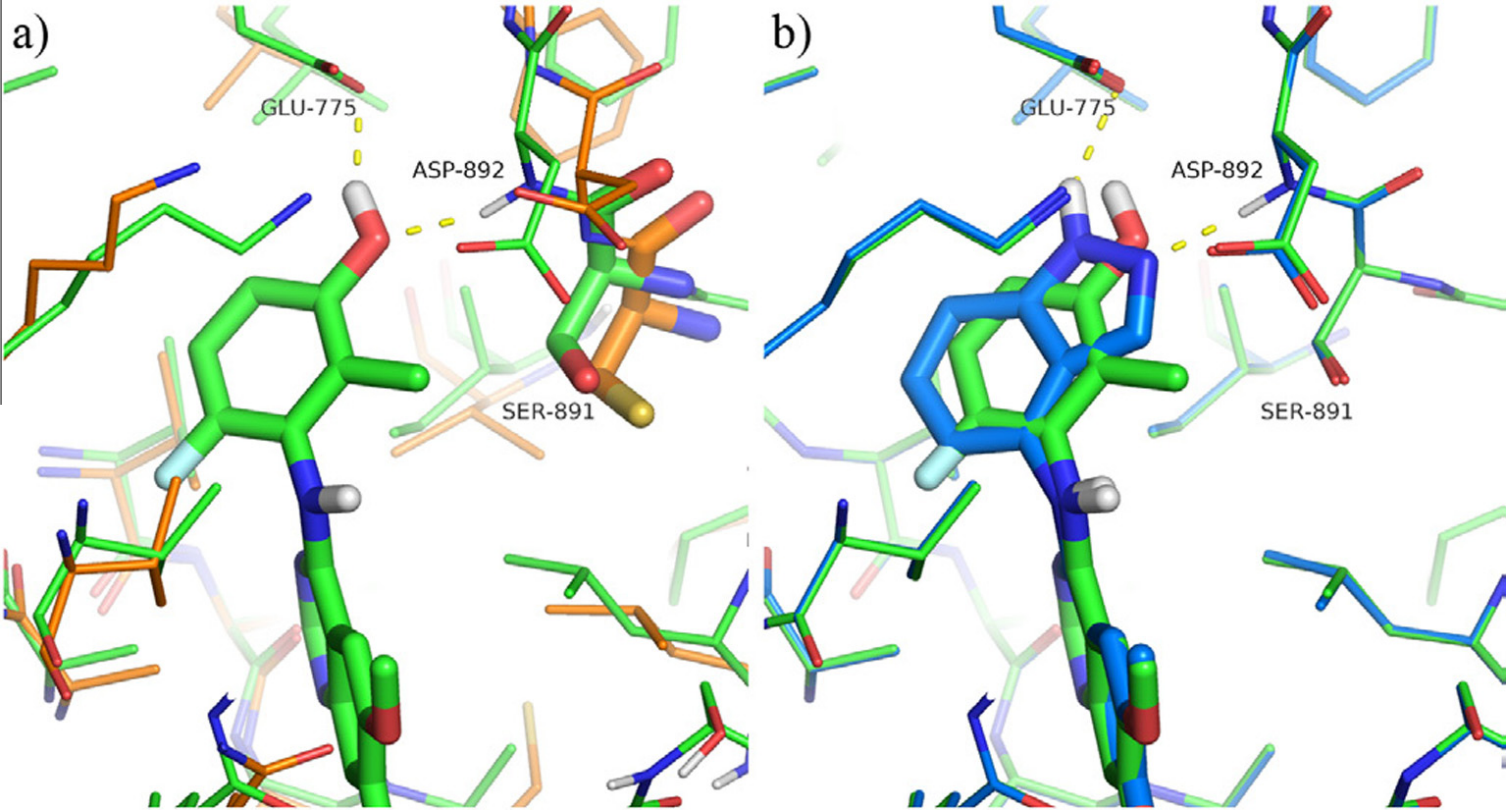
(a) Compound **7** modelled in RET (green carbon atoms, derived from PDB 5AMN^16^), compared with the aligned X-ray structure of KDR (PDB 3CJG, orange carbons (b) comparison of compounds **7** (green carbon atoms) and **17** (light blue carbon atoms) modelled in RET. Figure prepared using the Pymol Molecular Graphics System (Schrödinger, LLC, New York, NY).

**Figure 3:**
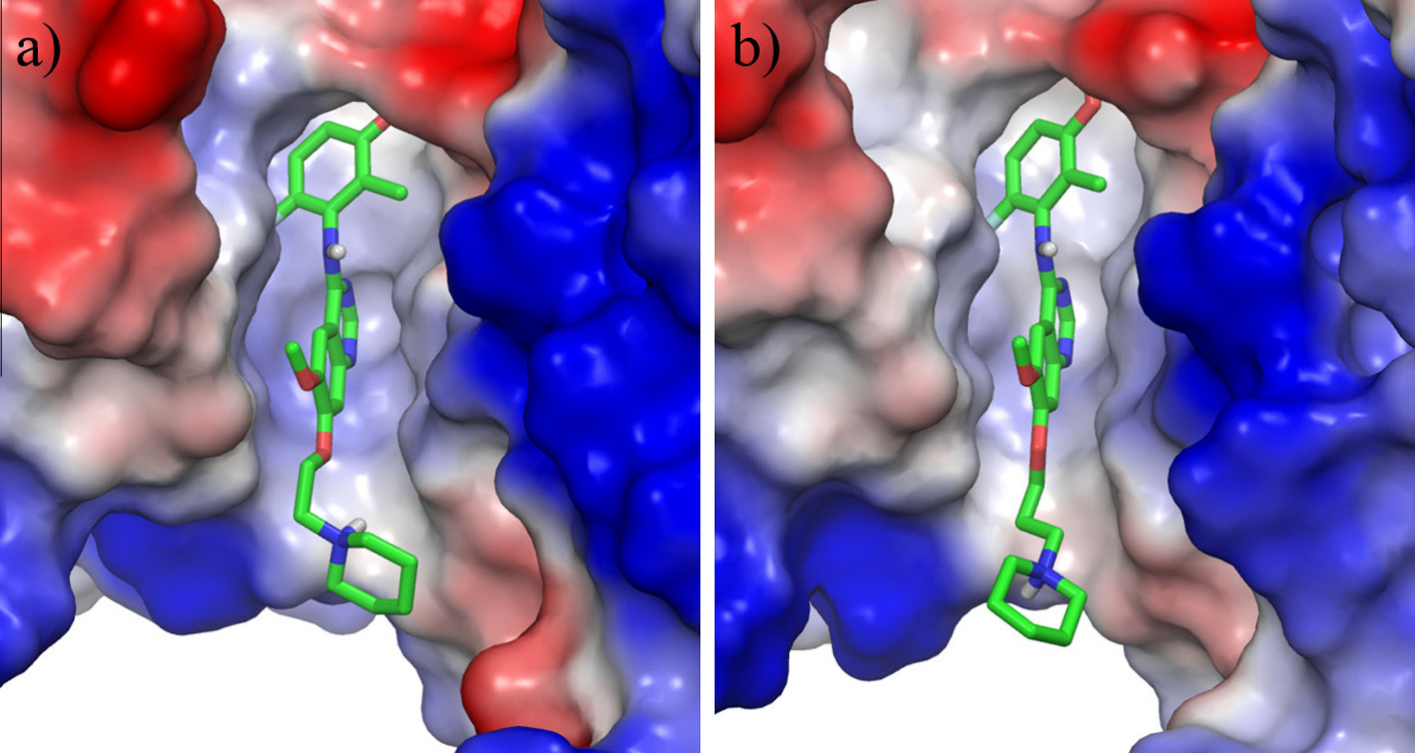
Representative snapshots of (a) **29** and (b) **30** bound to RET from molecular dynamics simulations, highlighting the location of the 7-substituent at the entrance to the ATP site. Protein solvent-accessible surfaces coloured by electrostatic potential (red = negative potential, blue = positive); water molecules and non-polar hydrogen atoms omitted for clarity. Figure prepared using the Pymol Molecular Graphics System (Schrödinger, LLC, New York, NY).

**Scheme 1:**
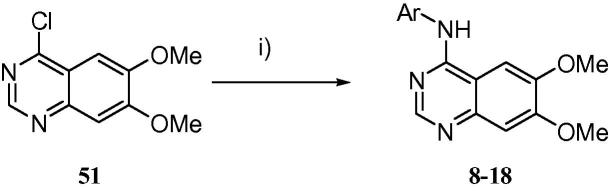
Preparation of bioisosteres **8**–**18**. Reagents and conditions: (i) aromatic amine, acetonitirile, microwave, 100 °C, 1 h, 11–93%.

**Scheme 2:**
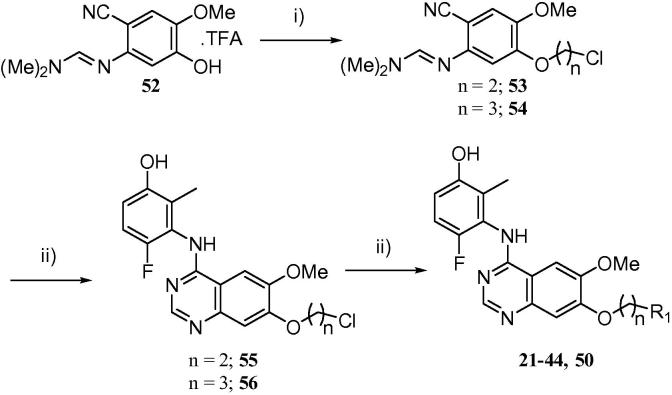
Synthesis of compounds **21**–**44**, **50**. Reagents and conditions: (i) bromochloroalkane, potassium carbonate, acetonitirile, 50–80 °C, 6 h, 61–63%; (ii) 3-amino-4-fluoro-2-methylphenol, acetic acid, 120 °C, 2 h, 24–48%; (iii) amine, microwave, 110 °C, 2–24 h, 5–87%.

**Scheme 3:**
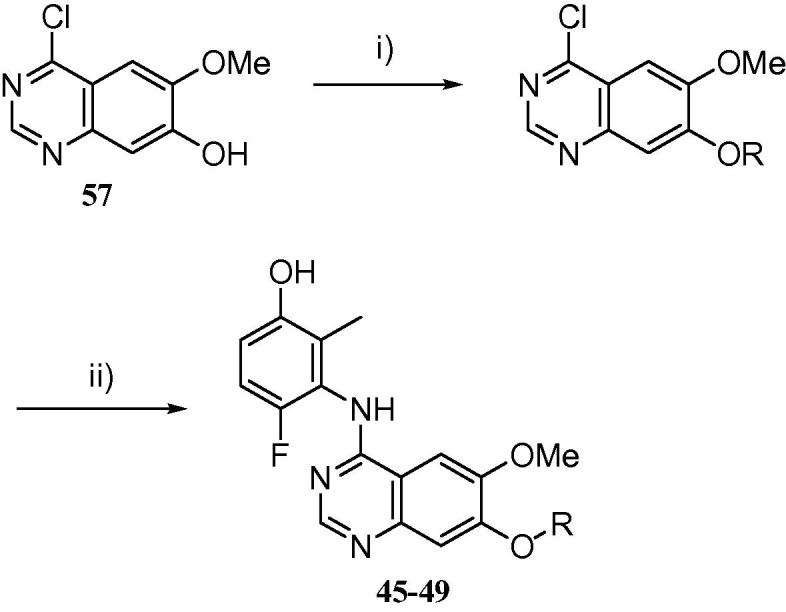
Synthesis of compounds **45**–**49**. Reagents and conditions: (i) alcohol, triphenylphosphine, DIAD, THF, 35 °C, 16 h, 33–91%; (ii) 3-amino-4-fluoro-2-methylphenol, acetic acid, 120 °C, 2 h, 11–67%.

**Table 1:** Biological data for exemplar bioisosteric replacements

**Figure:**
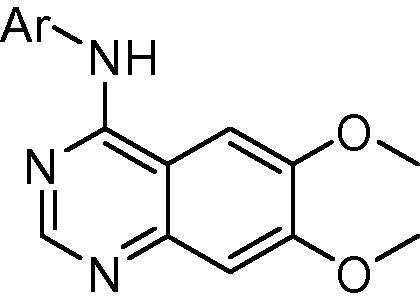

Compound	Ar	RET IC_50_^a^ (μM)	Enzymatic selectivity versus KDR	RET cellular IC_50_^a^ (μM)	KDR cellular IC_50_^a^ (μM)
**7**	2-Me-3-OH-6-F-Ph	0.044 (0.006)	130	2.10 (1.0)	>10
**8**	Ph	1.7 (0.29)	>5	n/d	n/d
**9**	3-MeSO_2_NH-Ph	7.7 (10)	n/d	>10	>10
**10**	3-CHF_2_-Ph	2.9 (0.3)	2	>10	>10
**11**	6-Indolyl	1.7 (0.48)	2	>10	>10
**12**	4-Indolyl	9.1 (1.1)	n/d	>10	>10
**13**	6-Benzimidazolyl	>30	n/d	>10	>10
**14**	4-Benzimidazolyl	4.3 (0.6)	3	n/d	n/d
**15**	7-Indazoloy	3.3 (0.19)	>30	n/d	n/d
**16**	6-Indazolyl	0.28 (0.01)	2	>10	5.2 (1.4)
**17**	5-Indazolyl	0.60 (0.18)	5	>10	>10
**18**	4-Indazolyl	0.14 (0.06)	38	4.6 (0.26)	>10

a Biological data are stated as the geometric mean of at least four independent determinations. Standard deviations are given in parentheses. n/d = not determined.

**Table 2:** Biological data for 7-alkoxy derivatives

**Figure:**
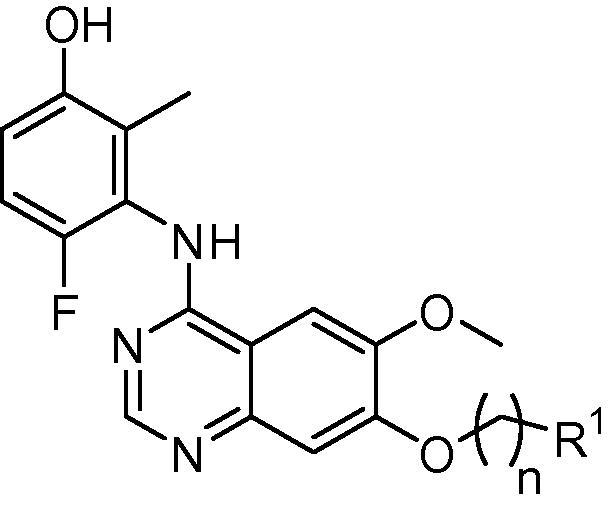

Compound	*n*	R^1^	RET IC_50_^a^ (μM)	Enzymatic selectivity versus KDR	RET cellular IC_50_^a^ (μM)	KDR cellular IC_50_^a^ (μM)
**7**	1	H	0.044 (0.006)	130	2.1 (1.0)	>10
**21**	2	NH_2_	0.13 (0.057)	57	>10	>10
**22**	3	NH_2_	0.048 (0.018)	110	>10	>10
**23**	2	NHMe	0.10 (0.01)	280	>10	>10
**24**	3	NHMe	0.023 (0.017)	320	6.5 (2.2)	>10
**25**	2	NMe_2_	0.35 (2.0)	80	>10	>10
**26**	3	NMe_2_	0.007 (0.001)	710	4.6 (0.08)	>10
**27**	2	*N*-Pyrrolidine	0.10 (0.02)	250	>10	>10
**28**	3	*N*-Pyrrolidine	0.015 (0.008)	290	>10	>10
**29**	2	*N*-Piperidine	0.12 (0.027)	215	>10	>10
**30**	3	*N*-Piperidine	0.015 (0.005)	340	5.1 (0.5)	>10
**31**	2	*N*-Piperazine	0.017 (0.002)	740	>10	>10
**32**	3	*N*-Piperazine	0.005 (0.001)	630	7.6 (1.9)	>10
**33**	2	*N*-Homopiperazine	0.065 (0.010)	270	6.7 (0.7)	>10
**34**	3	*N*-Homopiperazine	0.011 (0.005)	390	6.4 (0.35)	>10
**35**	2	*N*-Methylpiperazine	0.044 (0.01)	340	>10	>10
**36**	3	*N*-Methylpiperazine	0.029 (0.33)	210	4.6 (0.57)	>10
**37**	2	*N*-Morpholine	0.014 (0.009)	>125	>10	>10
**38**	3	*N*-Morpholine	0.011 (0.007)	380	4.0 (0.058)	>10
**39**	2	*N*-Thiomorpholine 1,1-dioxide	0.014 (0.0002)	650	>10	>10
**40**	3	*N*-Thiomorpholine 1,1-dioxide	0.009 (0.005)	>500	>10	>10
**41**	2	(3′-Fluoro)-*N*-pyrrolidine	0.030 (0.003)	105	1.3 (0.23)	>10
**42**	2	2′-CF_3_-*N*-pyrrolidine	0.15 (0.10)	205	2.1 (0.13)	>10
**43**	3	2′-CF_3_-*N*-pyrrolidine	0.062 (0.12)	120	0.6 (0.05)	>10
**44**	1	3′-Pyridyl	0.012 (0.002)	640	1.1 (0.05)	>10
**45**	1	2′-(*N*-Methylimidazole)	0.11 (0.03)	120	>10	>10
**46**	1	3′-(5-Methyl-1,2,4-oxadiazole)	0.024 (0.006)	190	5.9 (1.0)	>10
**47**	1	4′-(*N*-Methylpiperidine)	0.078 (0.012)	22	4.1 (0.38)	5.6
**48**	1	2′-Tetrahydrofuran	0.018 (0.007)	410	1.8 (0.06)	>10
**49**	2	OMe	0.014 (0.002)	270	1.2 (0.7)	>10
**50**	3	OMe	0.038 (0.009)	150	2.0 (0.3)	>10

a Biological data are stated as the geometric mean of at least four independent determinations. Standard deviations are given in parentheses.

**Table 3:** In vitro metabolic stability data for selected derivatives

Compound	Human microsomal half-life (min)	Human hepatocyte half-life (min)
**7**	220	66
**41**	58	160
**42**	4.7	—
**43**	2.7	—
**44**	32	60
**48**	50	83
**49**	2900	59
**50**	68	99
