# Supplementary material for: Anilinoquinazoline inhibitors of the RET kinase domain—Elaboration of the 7-position
Source: Bioorg Med Chem Lett. 2016 Jun 1;26(11):2724–9. doi: 10.1016/j.bmcl.2016.03.100 (PMC4896930; doi:10.1016/j.bmcl.2016.03.100)
Supplement: Supplementary data [file mmc1.docx]

# Anilinoquinazoline inhibitors of the RET kinase

# domain – elaboration of the 7-position.

**Supplementary Information.**

Allan M. Jordan, Habiba Begum, Emma Fairweather, Samantha Fritzl, Kristen Goldberg, Gemma V. Hopkins, Niall M. Hamilton, Amanda J. Lyons, H. Nikki March, Rebecca Newton, Helen F. Small, Swamy Vishwanath, Ian D. Waddell, Bohdan Waszkowycz, Amanda J. Watson and Donald J. Ogilvie.

***Contents.***

|  | Page |
| --- | --- |
| Analytical Data Summary for all Compounds | 2 |
| Experimental Procedures | 3 |
| Representative Synthetic Methodology | 5 |
| Biological Assay Protocols | 7 |
| Molecular Modelling Details | 8 |
| References | 9 |

**Analytical Data Summary for all Compounds**

| **COMPOUND** | **pH 4** | | | | **pH 10** | | | | **NMR**  **Purity** |
| --- | --- | --- | --- | --- | --- | --- | --- | --- | --- |
|  | **RT** | **Obs MW** | **Adduct** | **Purity** | **RT** | **Obs MW** | **Adduct** | **Purity** |  |
| **8** | 0.77 | 282.5 | [M+H]+ | >95 | 0.93 | 282.5 | [M+H]+ | >95 | 90-95% |
| **9** | 0.69 | 375.5 | [M+H]+ | >95 | 0.82 | 375.5 | [M+H]+ | >95 | 90-95% |
| **10** | 0.89 | 332.5 | [M+H]+ | >95 | 0.98 | 332.5 | [M+H]+ | >95 | >95% |
| **11** | 0.76 | 321.6 | [M+H]+ | >95 | 0.91 | 321.6 | [M+H]+ | >95 | 90-95% |
| **12** | 0.7 | 321.5 | [M+H]+ | >95 | 0.85 | 321.6 | [M+H]+ | >95 | >95% |
| **13** | 0.52 | 322.6 | [M+H]+ | >95 | 0.73 | 322.6 | [M+H]+ | >95 | 90-95% |
| **14** | 0.57 | 322.5 | [M+H]+ | >95 | 0.77 | 322.5 | [M+H]+ | >95 | 90-95% |
| **15** | 0.67 | 322.5 | [M+H]+ | >95 | 0.82 | 322.5 | [M+H]+ | >95 | 90-95% |
| **16** | 0.66 | 322.6 | [M+H]+ | >95 | 0.81 | 322.6 | [M+H]+ | >95 | 90-95% |
| **17** | 0.62 | 322.5 | [M+H]+ | >95 | 0.77 | 322.5 | [M+H]+ | >95 | 90-95 |
| **20** | 0.64 | 375.5 | [M+H]+ | >95 | 0.79 | 322.5 | [M+H]+ | >95 | >95% |
| **21** | 0.51 | 359.2 | [M+H]+ | >85 | 0.69 | 359.1 | [M+H]+ | 85-90 | 90-95 |
| **22** | 0.53 | 373.1 | [M+H]+ | 90-95 | 0.69 | 373.1 | [M+H]+ | <85 | 90-95 |
| **23** | 0.54 | No ion | n/a | >95 | 0.74 | 373.3 | [M+H]+ | 90-95 | 90-95 |
| **24** | 0.6 | No ion | n/a | >95 | 0.74 | 387.4 | [M+H]+ | >95 | 90-95 |
| **25** | 0.54 | 387.4 | [M+H]+ | >95 | 0.82 | 387.4 | [M+H]+ | >95 | >95 |
| **26** | 0.56 | 401.3 | [M+H]+ | >95 | 0.83 | 401.3 | [M+H]+ | >95 | 90-95 |
| **27** | 0.57 | 413.4 | [M+H]+ | >95 | 0.88 | 413.4 | [M+H]+ | >95 | 90-95 |
| **28** | 0.59 | 425.2 | [M-H]- | >95 | 0.88 | 425.2 | [M-H]- | >95 | 90-95 |
| **29** | 0.59 | 427.4 | [M+H]+ | >95 | 0.96 | 427.4 | [M+H]+ | >95 | >95 |
| **30** | 0.61 | 439.2 | [M-H]- | >95 | 0.95 | 439.2 | [M-H]- | >95 | 90-95 |
| **31** | 0.56 | 426.4 | [M-H]- | >95 | 0.72 | 426.4 | [M-H]- | 90-95 | 90-95 |
| **32** | 0.55 | 440.4 | [M-H]- | >95 | 0.75 | 442.5 | [M+H]+ | >95 | 90-95 |
| **33** | 0.63 | 441.4 | [M+H]+ | >95 | 1.00 | 441.5 | [M+H]+ | >95 | 90-95 |
| **34** | 0.64 | 455.4 | [M+H]+ | >95 | 1.03 | 455.4 | [M+H]+ | >95 | 90-95 |
| **35** | 0.56 | 442.7 | [M+H]+ | 90-95 | 0.78 | 442.7 | [M+H]+ | 85-90 | 85-90 |
| **36** | 0.56 | 456.3 | [M+H]+ | >95 | 0.78 | 456.4 | [M+H]+ | >95 | 90-95 |
| **37** | 0.56 | 429.4 | [M+H]+ | >95 | 0.79 | 429.3 | [M+H]+ | 90-95 | 90-95 |
| **38** | 0.57 | 441.2 | [M+H]+ | >95 | 0.83 | 441.2 | [M+H]+ | >95 | 90-95 |
| **39** | 0.69 | 477.4 | [M+H]+ | >95 | 0.77 | 477.3 | [M+H]+ | >95 | 90-95 |
| **40** | 0.69 | 491.4 | [M+H]+ | <85 | 0.81 | 491.4 | [M+H]+ | 85-90 | 90-95 |
| **41** | 0.57 | 445.2 | [M+H]+ | >95 | 0.90 | 445.2 | [M+H]+ | 90-95 | 90-95 |
| **42** | 0.98 | 481.4 | [M+H]+ | >95 | 1.08 | 481.3 | [M+H]+ | >95 | 90-95 |
| **43** | 0.99 | 495.3 | [M+H]+ | 90-95 | 1.12 | 495.4 | [M+H]+ | 90-95 | 90-95 |
| **44** | 0.71 | No ion | n/a | >95 | 0.83 | 407.6 | [M+H]+ | 85-90 | >95 |
| **45** | 0.56 | 410.3 | [M+H]+ | 90-95 | 0.78 | 410.3 | [M+H]+ | 90-95 | >95 |
| **46** | 0.75 | 412.6 | [M+H]+ | 90-95 | 0.75 | 412.6 | [M+H]+ | 90-95 | >95 |
| **47** | 0.55 | 425.3 | [M-H]- | 90-95 | 0.90 | 425.3 | [M-H]- | >95 | 90-95 |
| **48** | 0.78 | 400.3 | [M+H]+ | >95 | 0.90 | 400.4 | [M+H]+ | >95 | >95 |
| **49** | 0.73 | 374.5 | [M+H]+ | >95 | 0.83 | 374.5 | [M+H]+ | >95 | >95 |
| **50** | 0.79 | 388.5 | [M+H]+ | >95 | 0.89 | 388.5 | [M+H]+ | >95 | >95 |

**Experimental Procedures.**

Flash chromatography was performed using pre-packed silica gel cartridges (KP-Sil SNAP, Biotage, Hengoed UK or RediSep Rf, Isco). Thin layer chromatography was conducted with 5 × 10 cm plates coated with Merck Type 60 F_254_ silica gel to a thickness of 0.25 mm. All reagents obtained from commercial sources were used without further purification. Anhydrous solvents were obtained from the Sigma-Aldrich Chemical Company Ltd. or Fisher Chemicals Ltd., and used without further drying. HPLC grade solvents were obtained from Fisher Chemicals Ltd.

All compounds were > 90% purity as determined by examination of both the LC-MS and ^1^H NMR spectra unless otherwise indicated. Where Cl or Br were present, expected isotopic distribution patterns were observed.

^1^H NMR

Proton (^1^H) and carbon (^13^C) NMR spectra were recorded on a 300 MHz Bruker spectrometer. Solutions were typically prepared in either deuterochloroform (CDCl_3_), deuteromethanol (CD_3_OD) or deuterated dimethylsulfoxide (*d*^6^-DMSO) with chemical shifts referenced to tetramethylsilane (TMS) or deuterated solvent as an internal standard. ^1^H NMR data are reported indicating the chemical shift (*δ*), the integration (e.g. 1H), the multiplicity (s, singlet; d, doublet; t, triplet; q, quartet; m, multiplet; br, broad; dd, doublet of doublets etc.) and the coupling constant (*J*) in Hz (app implies apparent coupling on broadened signals). Deuterated solvents were obtained from the Sigma-Aldrich Chemical Company, Goss or Fluorochem.

Analytical LC-MS.

LC-MS analyses were performed on a Waters Acquity UPLC system fitted with BEH C18 1.7 μM columns (2.1 × 50 mm) and with UV diode array detection (210–400 nm). Positive and negative mass ion detection was performed using a Waters SQD detector. Analyses were performed with either buffered acidic or basic solvents and gradients as detailed below:

Low pH:

Solvent A – Water + 10mM ammonium formate + 0.1% formic acid
Solvent B – Acetonitrile + 5% water + 0.1% formic acid

High pH:

Solvent A – Water + 10mM ammonium hydrogen carbonate + 0.1% ammonia solution
Solvent B – Acetonitrile + 0.1% ammonia solution

Gradient:

| Time | Flow rate (mL/min) | % Solvent A | % Solvent B |
| --- | --- | --- | --- |
| 0 | 0.6 | 95 | 5 |
| 1.2 | 0.6 | 5 | 95 |
| 1.7 | 0.6 | 5 | 95 |
| 1.8 | 0.6 | 95 | 5 |

Preparative HPLC

Some compounds were purified by preparative HPLC on a Waters FractionLynx MS autopurification system, with a Waters XBridge 5 µm C18, 100 mm × 19 mm i.d. column, running at a flow rate of 20 mL/min with UV diode array detection (210–400 nm) and mass-directed collection using both positive and negative mass ion detection.

Purifications were performed using buffered acidic or basic solvent systems as appropriate. Compound retention times on the system were routinely assessed using a 30-50 μL test injection and a standard gradient, then purified using an appropriately chosen focussed gradient as detailed below, based upon observed retention time.

Low pH:

Solvent A – Water + 10mM ammonium formate + 0.1% formic acid
Solvent B – Acetonitrile + 5% water +0.1% formic acid

High pH:

Solvent A – Water + 10mM ammonium formate + 0.1% ammonia solution
Solvent B – Acetonitrile + 5% water + 0.1% ammonia solution

Standard Gradient:

| Time | Flow rate (mL/min) | % Solvent A | % Solvent B |
| --- | --- | --- | --- |
| 0 | 20 | 90 | 10 |
| 0.3 | 20 | 90 | 10 |
| 8.5 | 20 | 2 | 98 |
| 12 | 20 | 2 | 98 |
| 12.5 | 0 | 2 | 98 |

Focussed Gradients:

|  |  | % Solvent B | | | | |
| --- | --- | --- | --- | --- | --- | --- |
|  |  | Retention time on standard gradient (min.) | | | | |
| Time | Flow rate (mL/min) | 0 – 5.2 | 4.9 – 6.6 | 6.3 – 7.5 | 7.3 – 9.5 | 9.3 - 12 |
| 0 | 20 | 10 | 10 | 10 | 10 | 10 |
| 0.25 | 20 | 10 | 10 | 10 | 10 | 10 |
| 0.35 | 20 | 10 | 20 | 35 | 45 | 60 |
| 10 | 20 | 45 | 55 | 65 | 75 | 98 |
| 12 | 20 | 98 | 98 | 98 | 98 | 98 |
| 12.5 | 0 | 98 | 98 | 98 | 98 | 98 |

**Representative synthetic methodology.**

***Scheme 1:***

***N*-(1*H*-Indazol-6-yl)-6,7-dimethoxy-3,4-dihydroquinazolin-4-amine 16**

4-Chloro-6,7-dimethoxy-quinazoline (100 mg, 0.4500 mmol) and 1*H*-indazol-6-amine (59.27 mg, 0.4500 mmol) were mixed in MeCN (2 mL) and irradiated in a CEM microwave at 100 ^o^C for one hour. The reaction mixture was allowed to cool and then passed through a short pad of silica. After concentration of the filtrate, the resultant solid was washed sequentially with acetonitrile and diethyl ether and dried *in vacuo* to give **16** (0.13g, 81%) as a light brown solid. ^1^H NMR (DMSO-*d*_6_) δ 13.21 (br s, 1H), 11.37 (s, 1H), 8.84 (s, 1H), 8.30 (s, 1H), 8.12 (s, 1H), 7.94 (s, 1H), 7.85 (d, *J*=8.6 Hz, 1H), 7.41 (d, *J*=8.6 Hz, 1H), 7.34 (s, 1H), 4.03 (s, 3H), 4.01 (s, 3H)

***Scheme 2:***

***E*-*N*’-(5-(3-Chloropropoxy)-2-cyano-4-methoxyphenyl)-*N,N*-dimethylformimidamide 54**

To solution of **52**^1^ (7.8g, 24.6 mmol) in acetonitrile (75 mL) was added potassium carbonate (16.9g, 123 mmol) and the mixture heated at 50 ^o^C for 3 h. 1-Bromo-3-chloropropane (7.7g, 49 mmol) was added and the mixture then heated at 110 ^o^C for 3 h. After the reaction was complete by TLC (50% ethyl acetate in hexane) the mixture was cooled to room temperature, filtered through a pad of Celite and washed with ethyl acetate. The filtrate obtained was concentrated *in vacuo* resulting in crude product which was purified by crystallization using THF:hexane to afford **54** (6.7g, 63%). ^1^H NMR (DMSO-*d*_6_) δ 7.91 (s, 1 H), 7.10 (s, 1 H), 6.77 (s, 1 H), 4.15 (t, *J*=6.1 Hz, 2 H), 3.78 (t, *J*=6.5 Hz, 2 H), 3.73 (s, 3 H), 3.05 (s, 3 H), 2.96 (s, 3 H), 2.19 (quin, *J*=6.1 Hz, 2 H).

**3-((7-(3-Chloropropoxy)-6-methoxyquinazolin-4-yl)amino)-4-fluoro-2-methylphenol 56.**

To a solution of **54** (4g, 13.5 mmol) in acetic acid (12 mL) at room temperature was added 2-methyl-3-hydroxy-6-fluoroaniline (2.3g, 16 mmol). After addition, the mixture was refluxed at 120 ^o^C for 3 h. After the reaction was complete by TLC (10% methanol in CHCl_3_), the mixture was concentrated *in vacuo* and twice azeotroped with toluene. Flash column chromatography, eluting with 1-3% MeOH in DCM gave **56** (1.3g, 24%). ^1^H NMR (DMSO-*d*_6_) δ 9.38 (s, 1 H), 9.25 (s, 1 H), 8.25 (s, 1 H), 7.85 (s, 1 H), 7.17 - 7.22 (m, 1 H), 6.94 (t, *J*=9.2 Hz, 1 H), 6.76 (dd, *J*=9.0, 4.5 Hz, 1 H), 4.26 (t, *J*=5.8 Hz, 2 H), 3.94 (s, 3 H), 3.82 (t, *J*=6.5 Hz, 2 H), 2.22 - 2.29 (m, 2 H), 1.98 (s, 3 H).

**General procedure for compounds 21-44 and 50.**

To a 5 mL microwave tube was added **56** (1eq, 100 mg), the relevant amine (10eq) and DMF (10 volume equivalents). The tube was sealed and heated to 110 ^o^C for 2-24 h. On consumption of starting materials by LCMS, the reaction was concentrated *in* *vacuo* and purified by preparative HPLC.

**4-Fluoro-3-((6-methoxy-7-(3-(2-(trifluoromethyl)pyrrolidin-1-yl)propoxy)quinazolin-4-yl)amino)-2-methylphenol 43.**

Prepared as above using 2-(trifluoromethyl)pyrrolidine to give **43** (14mg, 11.1%) as a light brown solid. ^1^H NMR (CD_3_OD): δ 8.23 (s, 1 H), 7.72 - 7.77 (m, 1 H), 7.16 (s, 1 H), 6.87 - 6.92 (m, 1 H), 6.79 (d, *J* = 8.08, 1 H), 4.25 (t, *J*=6.32 Hz, 2 H), 4.03 (s, 3 H), 3.35 (m, 1 H), 3.21 - 3.25 (m, 2 H), 3.09 - 3.18 (m, 2 H), 2.77 - 2.83 (m, 2 H), 2.38 - 2.53 (m, 2 H), 2.09 - 2.14 (s, 3 H), 1.86 - 1.95 (m, 2 H).

***Scheme 3:***

**4-Chloro-6-methoxy-7-(2-methoxyethoxy)quinazoline.**

Polymer-bound triphenylphosphine (~1.6mmol/g, 500 mg, 1.9 mmol) was stirred for 5 minutes in DCM (5 mL). 4-Chloro-6-methoxy-quinazolin-7-ol **57**^2,3^ (150 mg, 0.71 mmol) in acetonitrile (2 mL), methoxyethanol (0.06 mL, 0.71 mmol) and diisopropyl azodicarboxylate (0.18 mL, 0.93 mmol) were added to the solution and the reaction was stirred at room temperature for 16 hours. The mixture was then filtered and the beads washed with MeCN (20 mL) and DCM (20 mL). Concentration *in vacuo* and purification by flash chromatography using a gradient of 0 - 100% EtOAc in hexane gave product, partially contaminated with reduced DIAD. This material was slurried in ether (20 mL) and filtered through Celite to give 4-chloro-6-methoxy-7-(2-methoxyethoxy)quinazoline (105 mg, 0.37 mmol, 52%) as a white solid. 1H NMR (DMSO-*d*_6_): δ 8.88 (s, 1H), 7.49 (s, 1H), 7.41 (s, 1H), 4.36 (m, 2H), 4.01 (s, 3H), 3.76 (m, 2H), 3.33 (s, 3H).

**4-Fluoro-3-((6-methoxy-7-(2-methoxyethoxy)quinazolin-4-yl)amino)-2-methylphenol** **49.**

A suspension of 3-amino-4-fluoro-2-methyl-phenol (18.9 mg, 0.13 mmol), 4-chloro-6-methoxy-7-(2-methoxyethoxy)quinazoline (30 mg, 0.11 mmol) and 5-6 N HCl in IPA (0.02 mL, 0.11 mmol) in IPA (2 mL) was heated at 100 °C for 30 mins in the microwave. The reaction was evaporated to dryness and the residues partitioned between ethyl acetate and saturated aqueous sodium bicarbonate. The organic phase was separated, washed with brine and concentrated *in vacuo*. The crude material was purified by flash chromatography eluting with a gradient of 0 - 10% MeOH in DCM to give **49** (6 mg, 0.0157 mmol, 14%) as an off-white powder. ^1^H NMR: (DMSO-*d*_6_): δ 9.40 (s, 1H), 9.25 (s, 1H), 8.25 (s, 1H), 7.85 (s, 1H), 7.19 (s, 1H), 6.95 (t, J = 9.2 Hz, 1H), 6.77 (dd, J = 9.0 and 4.3 Hz, 1H), 4.26 (m,, 2H), 3.94 (s, 3H), 3.47 (m, 2H), 3.34 (s, 3H), 1.99 (s, 3H).

**Biological Assay Protocols.**

**Biochemical assay.**

Kinase activity was detected using CisBio HTRF kinEASE kit based on time-resolved fluorescence transfer (FRET). The assay was performed in 384-well white plates (Corning #3574) in a reaction volume of 10 µL containing 1X Cisbio enzymatic buffer supplemented with a final concentration of 5 mM MgCl_2_, 1 mM DTT, 10 nM SEB and 0.01% Triton X100 for RET. The same buffer was used for the KDR biochemical assay with the addition of 2 mM MnCl_2_.

Inhibitors were pre-incubated in the plate for 15 minutes with 5 µL kinase and assay buffer at the following concentrations; 13 pM RET (Carna Biosciences; 08-159) and 150 pM KDR (Millipore; 14-630). The reaction was initiated by the addition of 5 µL ATP and substrate at 2X final reaction concentrations. For RET, this was 18 µM and 2 µM; for KDR, this was 16 µM and 1 µM, respectively. Reactions were performed at ATP Km for each target. The assay was allowed to proceed at room temperature for 20 minutes before terminating with the addition of 10 µL HTRF detection buffer containing EDTA supplemented with TK-antibody labelled with Eu3+-Cryptate (1:100 dilution) and streptavidin-XL665 (128 nM). Following incubation at room temperature for 1 hour, FRET signal was measured using the Pherastar FS Microplate Reader.

**BaF3 cellular assay**

The system originally developed by Daley and Baltimore^4^ was used, whereby IL3-dependent Ba/F3 cells are modified to express an activated recombinant kinase. Following removal of IL3, the modified cells are dependent on the activity of the recombinant kinase for survival and proliferation. Ba/F3 cell lines, harbouring KIF5B-RET (gift from Pasi Janne^5^) and KDR (Advanced Cellular Dynamics, San Diego) were maintained in RPMI-1640 media containing 10% FBS and appropriate antibiotics. Non-modified Ba/F3 cells (WT) were maintained in RPMI-1640 media containing 10% FBS and supplemented with 10 ng/mL recombinant mouse IL3 (R&D systems). For assessment of compound IC_50_, cells were plated into 384-well plates at 1500 or 3000 cells per well in 30 µL culture medium and compounds dispensed using an acoustic liquid handling platform (Labcyte, Sunnyvale, CA). Following incubation of the cells for 48 hours at 37 °C in a humidified 5% CO_2_ atmosphere, viability was determined by addition of 10 µL CellTiter-Glo reagent (Promega) and measurement of luminescence.

**Molecular Modelling Details**

The previously reported X-ray structure of a 3’-OH anilinoquinazoline complexed in RET (PDB 5AMN^6^) was the basis for protein-ligand docking and molecular dynamics studies using the Schrödinger modelling suite (2015 release). The Maestro Protein Preparation Wizard^7^ was used for initial processing of the X-ray structure, using default settings for protonation and geometry refinement. Models of **7** and **18** bound to RET (Figure 2) were generated by standard Glide SP^8^ docking to the dry protein model, followed by a constrained molecular mechanics refinement in Prime^9^ using the OPLS2005 forcefield with implicit MM-GBSA solvation, freezing the protein coordinates beyond a 5 Ångstrom shell around the ligand. Models of **29** and **30** bound to RET (Figure 3) were derived from unconstrained 4.8 ns molecular dynamics simulations in an explicit solvent model using Desmond^10^. Following initial Glide docking, default set-up protocols in Desmond were applied to solvate the protein-ligand complex, followed by default equilibration and production molecular dynamics simulations.

**References.**

1. Mortlock, A.A.; Patent application WO2004/058752.
2. Heron, N.M.; Pasquet, G.R.; Mortlock, A.A.; Jung, F. H. Patent application no. WO2004/094410.
3. Tasler, S.; Müller, O.; Weiber, T.; Herz, T.; Pegoraro, S.; Saeb, W.; Lang, M.; Krauss, R.; Totzke, F.; Zirrgiebel, U.; Ehlert, J.E.; Kubbutat, M.H.G.; Schächtele, C.; Bioorg. Med. Chem. Lett. 2009, 17, 6728.
4. Daley, G.Q.; Baltimore, D.; Proc. Natl. Acad. Sci. 1988, 85, 9312.
5. Lipson, D.; Capelletti, M.; Yelensky, R.; Otto, G.; Parker, A.; Jarosz, M.; Curran, J.A.; Balasubramanian, S.; Bloom, T.; Brennan, K.W.; Donahue, A.; Downing, S.R.; Frampton, G.M.; Garcia, L.; Juhn, F.; Mitchell, K.C.; White, E.; White, J.; Zwirko, Z.; Peretz, T.; Nechushtan, H.; Soussan-Gutman, L.; Kim, J.; Sasaki, H.; Kim, H.R.; Park, S.; Ercan, D, Sheehan, C.E.; Ross, J.S.; Cronin, M.T.; Jänne, P.A.; Stephens, P.J.; Nature Medicine 2012, 12, 382.
6. Newton, R.; Bowler, K.A.; Burns, M.; Chapman, P.J.; Fairweather, E.E.; Fritzl, S.J.R.; Goldberg, K.M.; Hamilton, N.M.; Holt, S.V.; Hopkins, G.V.; Jones, S.D.; Jordan, A.M.; Lyons, A.J.; March, H.N.; McDonald, N.Q.; Maguire, L.A.; Mould, D.P.; Purkiss, A.G.; Small, H.F.; Stowell, A.I.J.; Thomson, G.J.; Waddell, I.D.; Waszkowycz, B.; Watson, A.J.; Ogilvie, D.J. Eur. J. Med. Chem. 2016, 112, 20.
7. **Schrödinger Release 2015-3**: Schrödinger Suite 2015-3 Protein Preparation Wizard; Epik version 3.3, Schrödinger, LLC, New York, NY, 2015; Impact version 6.8, Schrödinger, LLC, New York, NY, 2015; Prime version 4.1, Schrödinger, LLC, New York, NY, 2015.
8. **Small-Molecule Drug Discovery Suite 2015-3**: Glide, version 6.8, Schrödinger, LLC, New York, NY, 2015.
9. **Schrödinger Release 2015-3**: Prime, version 4.1, Schrödinger, LLC, New York, NY, 2015.
10. **Schrödinger Release 2015-3**: Desmond Molecular Dynamics System, version 4.3, D. E. Shaw Research, New York, NY, 2015. Maestro-Desmond Interoperability Tools, version 4.3, Schrödinger, New York, NY, 2015.
